# Development of 3-dimensional printed simulation surgical training models for endoscopic endonasal and transorbital surgery

**DOI:** 10.3389/fonc.2022.966051

**Published:** 2022-08-05

**Authors:** Won-Jae Lee, Yong Hwy Kim, Sang-Duk Hong, Tae-Hoon Rho, Young Hoon Kim, Yun-Sik Dho, Chang-Ki Hong, Doo-Sik Kong

**Affiliations:** ^1^ Department of Neurosurgery, Samsung Medical Center, Sungkyunkwan University School of Medicine, Seoul, South Korea; ^2^ Department of Neurosurgery, Seoul National University Hospital, Seoul National University School of Medicine, Seoul, South Korea; ^3^ Department of Otorhinolaryngology—Head & Neck Surgery, Samsung Medical Center, Sungkyunkwan University School of Medicine, Seoul, South Korea; ^4^ Department of Neurosurgery, Ajou University Hospital, Ajou University School of Medicine, Suwon, South Korea; ^5^ Department of Neurosurgery, Asan Medical Center, University of Ulsan College of Medicine, Seoul, South Korea; ^6^ Department of Neurosurgery, Chungbuk National University Hospital, Chungbuk National University College of Medicine, Cheongju, South Korea

**Keywords:** 3-dimensional printed simulation, endoscopic endonasal approach, endoscopic transorbital approach, endoscopic skull base surgery, surgical simulation

## Abstract

**Background:**

Endoscopic skull base surgery (ESBS) is complex, requiring methodical and unremitting surgical training. Herein, we describe the development and evaluation of a novel three-dimensional (3D) printed simulation model for ESBS. We further validate the efficacy of this model as educational support in neurosurgical training.

**Methods:**

A patient-specific 3D printed simulation model using living human imaging data was established and evaluated in a task-based hands-on dissection program. Endoscopic endonasal and transorbital procedures were simulated on the model by neurosurgeons and otorhinolaryngology surgeons of varying experience. All procedures were recorded using a high-definition camera coupled with digital video recorder system. The participants were asked to complete a post-procedure questionnaire to validate the efficacy of the model.

**Results:**

Fourteen experts and 22 trainees participated in simulations, and the 32 participants completed the post-procedure survey. The anatomical realism was scored as 4.0/5.0. The participants rated the model as helpful in hand-eye coordination training (4.7/5.0) and improving surgical skills (4.6/5.0) for ESBS. All participants believed that the model was useful as educational support for trainees (4.7 [ ± 0.5]). However, the color (3.6/5.0) and soft tissue feedback parameters (2.8/5) scored low.

**Conclusion:**

This study shows that high-resolution 3D printed skull base models for ESBS can be generated with high anatomical accuracy and acceptable haptic feedback. The simulation program of ESBS using this model may be supplemental or provide an alternative training platform to cadaveric dissection.

## Introduction

Endoscopic skull base surgeries (ESBS), such as endoscopic endonasal (EES) and transorbital surgery (ETOS), are emerging as mainstream skull base surgeries and provide a minimally invasive treatment option for various skull base diseases (1–3). The skull base is a complex and unique anatomical structure, containing many critical neurovascular structures (4). High anatomical knowledge and experienced surgical techniques are imperative, which can only be obtained by unremitting surgical training. However, traditional surgical training for ESBS has been limited to cadaveric studies as there are currently no alternative training tools available prior to carrying out live surgeries (5, 6). The importance of hands-on training is overemphasized in ESBS as ESBS techniques require distinct ergonomics, equipment, and surgical practice, which are quite different from those of conventional transcranial microscopic skull base surgery (7). Encouragingly, recent advances in three-dimensional (3D) printing technology have allowed diverse simulation models for EES procedures (8–12). Such models have been used in medical education and surgical skill training as precise replications of the complex anatomical structures of the skull base (10, 13). The development of patient-specific models and improvement of cost-effectiveness have allowed these models to be used in preoperative planning and patient counseling (8). However, to the best of our knowledge, there are currently no simulation models for ETOS procedures. The pioneers of ETOS have demonstrated novel surgical techniques for ETOS procedures based on cadaveric studies and case descriptions (14–21). A steep learning curve, technical expertise, and unfamiliar anatomy limit the opportunities for surgical training of the ETOS procedure. Well-established 3D printed simulators offering good tactile sensation and realistic anatomy may supplement surgical training on a wider scale in a field where access to cases is limited.

We have designed a patient-specific 3D printed simulation model for surgical training of ESBS procedures, including EES and ETOS. The detailed anatomical information from multi-modal neuroimaging profiles was combined and reconstructed to identify the bony structures, soft tissues, vessels, and nerves of the skull base. We obtained preliminary application results and validated this 3D printed simulation model as an alternative training tool to cadaver dissection for EES and ETOS.

## Materials and methods

### Image acquisition

This study utilized living human medical imaging data, including computed tomography (CT) and magnetic resonance imaging (MRI) scans. A Lightspeed VCT CT scanner (GE Healthcare, Chicago, Illinois, United States) was used for continuous axial tomography of the skull with 0.625-mm slice thickness and 0.35-mm in-plane resolution, scanning parameters of 120 kV and 205.50 mAs, and a scanning matrix of 512 × 512 in size. MRI scans of the corresponding region were acquired using a 3.0T unit (Ingenia 3.0T CX; Philips, Amsterdam, Netherlands). Post-contrast T1-weighted axial images (1-mm-thick slices) were merged with CT images for a better bone-soft tissue contrast. Imaging data, including CT data of the skull and cervical vertebrae, MRI of tumors, and CT angiography of the intracranial arteries, were obtained, saved in the Digital Imaging and Communications in Medicine format, and exported.

### 3D printing processing

Using the collected imaging data, 3D reconstruction was performed using a dedicated software program (MEDIP PRO, Medical IP, Seoul, Republic of Korea). After the acquisition of 3D stereolithography image files suitable for printing, the physical model was printed on a 1:1 scale using 3D printing equipment. The model replicated the patient-specific anatomical structure for realistic visualization. The skull, brain, dura mater, cerebral venous sinuses, and arterial anatomies were segmented from the surrounding structures according to a grayscale, and tissues with a certain level of gray were converted into 3D images using the software algorithm. In this study, the prototype was obtained by a PolyJet process for additive manufacturing on J850 Prime and J750 Digital Anatomy Printing printers (Stratasys, Eden Prairie, MN).

### Model evaluation and application

**Figure 1:**
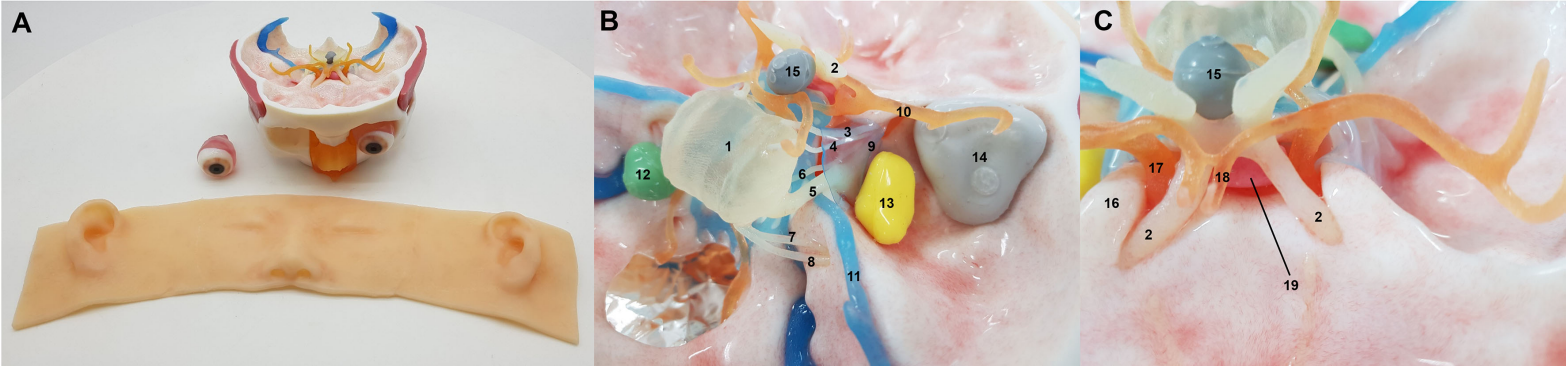
The three-dimensional printed simulation model. External view **(A)** and reconstructed neurovascular structures and lesions **(B, C)**. Key: 1, pons; 2, optic nerve; 3, oculomotor nerve; 4, trochlear nerve; 5, trigeminal nerve; 6, abducens nerve, 7, facial nerve; 8, vestibulocochlear nerve; 9, lateral wall of cavernous sinus; 10, middle cerebral artery; 11, superior petrosal sinus; 12, vestibular schwannoma lesion; 13, trigeminal schwannoma lesion; 14, spheno-orbital meningioma lesion; 15, craniopharyngioma lesion; 16, anterior clinoid process; 17, internal carotid artery; 18, anterior cerebral artery; 19, pituitary gland.

The main structures printed by the 3D printer were evaluated by five neurosurgeons with at least 5 years of working experience in performing ESBS. We confirmed the lesions’ locations, directions, sizes, and their spatial relationship with the adjacent neurovascular structures. Different surgical approaches were tested on the models to elaborate the virtual simulation. The models were observed and investigated at various endoscopic surgical angles. The final simulation models were established after repeated revision to replicate the conditions of an actual surgery (**Figure 1**).

On September 30, 2021, the first endoscopic skull base hands-on course using the 3D printed simulation model was held at Medtronic Innovation Center in Osong, Republic of Korea. Thirty-six neurosurgeons and ENT surgeons attended the course, and a total of 11 sets of simulation models were evaluated. The simulation procedures of the course included EES and ETOS procedures. Each procedure was demonstrated by the four experienced instructors. The model was connected to a neuronavigation system to aid in the accurate dissection. All procedures were recorded using a high-definition camera coupled with digital video recorder system. Subsequently, the other 32 participants were asked to complete a series of ESBS procedures using the simulation models. The participants were assigned to the expert or trainee group based on their previous exposure to ESBS. Experts were defined as surgeons with at least 5 years of experience in ESBS as the primary surgeon. All others were designated as the trainee group. The 3D printed anatomical structures and lesions corresponding to real patient-specific anatomy were approached and handled in each model.

### Evaluation survey

Immediately following the completion of the ESBS procedures, the participants were asked to complete a de-identified survey on their experience. The questionnaires were grouped into the following four domains: anatomical realism, task-based usefulness, usefulness as a training tool, and overall reactions. Likert scale questionnaires were administered to evaluate the anatomical and haptic accuracies of the simulator using the different instruments of dissection. The answers were scaled using a 5-point system, varying from strongly agree (5), agree (4), neutral (3), disagree (2), to strongly disagree (1).

### Ethical approval

The protocol for this study was reviewed and approved by the Ethics Committee of Samsung Medical Center.

## Results

A total of 32 (10 experts and 22 trainees) participants evaluated the models and completed the survey (**Table 1**). The four instructors with conflicts of interest were excluded from the survey. Endoscopic procedures were performed using a rigid 4-mm-diameter endoscope with 0°, 30°, and 45° lenses (Karl Storz). Twenty-four participants had at least one experience of a cadaver dissection course for ESBS.

**Table 1:** Baseline characteristics of participants who completed the hands-on endoscopic skull base surgery course and post-simulation questionnaire.

Features	Number (%)
Participants	32 (100)
Training grade	
Experts	10 (31)
Trainee	22 (69)
Male : Female	28:4
Age group	
30s	15 (47)
40s	15 (47)
50s	2 (6)
ESBS dissection course experience	
None	8 (25)
1	3 (10)
2	2 (6)
3≤	19 (59)

ESBS, endoscopic skull base surgery.

### EES procedures

**Figure 2:**
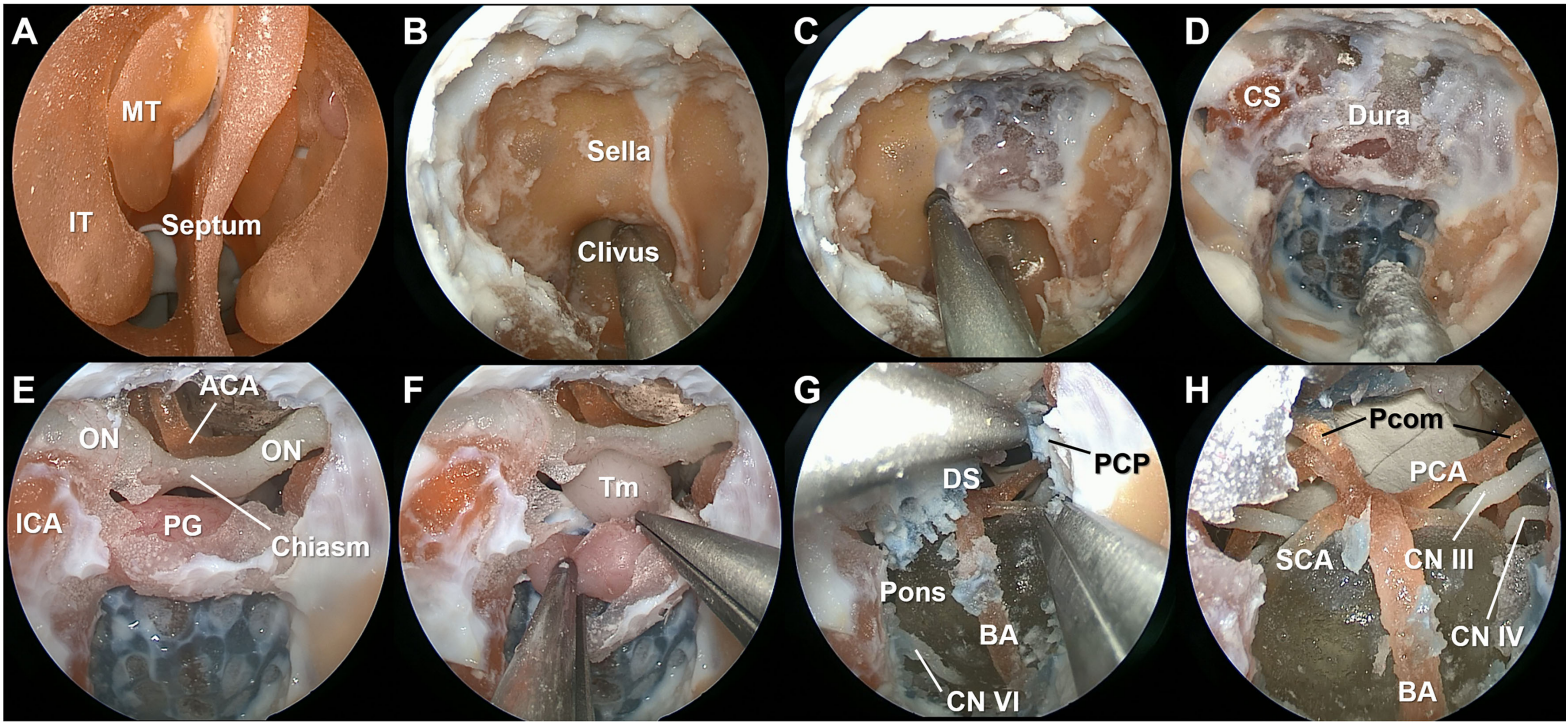
Stepwise dissection of the endoscopic endonasal surgery procedures using the simulation model. The mucosa and bony structures of the intranasal cavity were identified **(A)**. After wide sphenoidotomy **(B)**, the anterior skull base was drilled to expose the sella, clivus, and anterior wall of the cavernous sinus **(C, D)**. The dura ventral to the brain stem was colored in blue to indicate the basilar venous plexus. Intradural neurovascular structures were observed after opening the dura **(E)**, and the tumor lesion reconstructed from the patient with a stalk originated craniopharyngioma was identified **(F)**. After resection of the dorsum sella and posterior clinoid process, neurovascular structures within the posterior fossa were identified **(G, H)**. IT, inferior turbinate; MT, middle turbinate; ON, optic nerve; ICA, internal carotid artery; ACA, anterior cerebral artery; PG, pituitary gland; BA, basilar artery; Pcom, posterior communicating artery; PCA, posterior cerebral artery; SCA, superior cerebellar artery; CN, cranial nerve.

A standard bi-nostril transsphenoidal approach was performed using the simulation model (**Figure 2**, **Video 1**) The extent of dissection was tailored according to the practicality of the simulation model. The anatomy of the intranasal cavity was identified and resected to access the sphenoidal sinus. After entering the sphenoid sinus, the anterior skull base was drilled, and the dura of the sella, clivus, and anterior wall of the cavernous sinus (CS) were exposed. The dura was opened, and the intradural structures were explored. The model contained a tumor reconstructed from a patient with a craniopharyngioma. The feasibility of the transclival approach was also evaluated. After resection of the dorsum sella and posterior clinoid process, neurovascular structures ventral to the brain stem were identified.

### ETOS procedures

**Figure 3:**
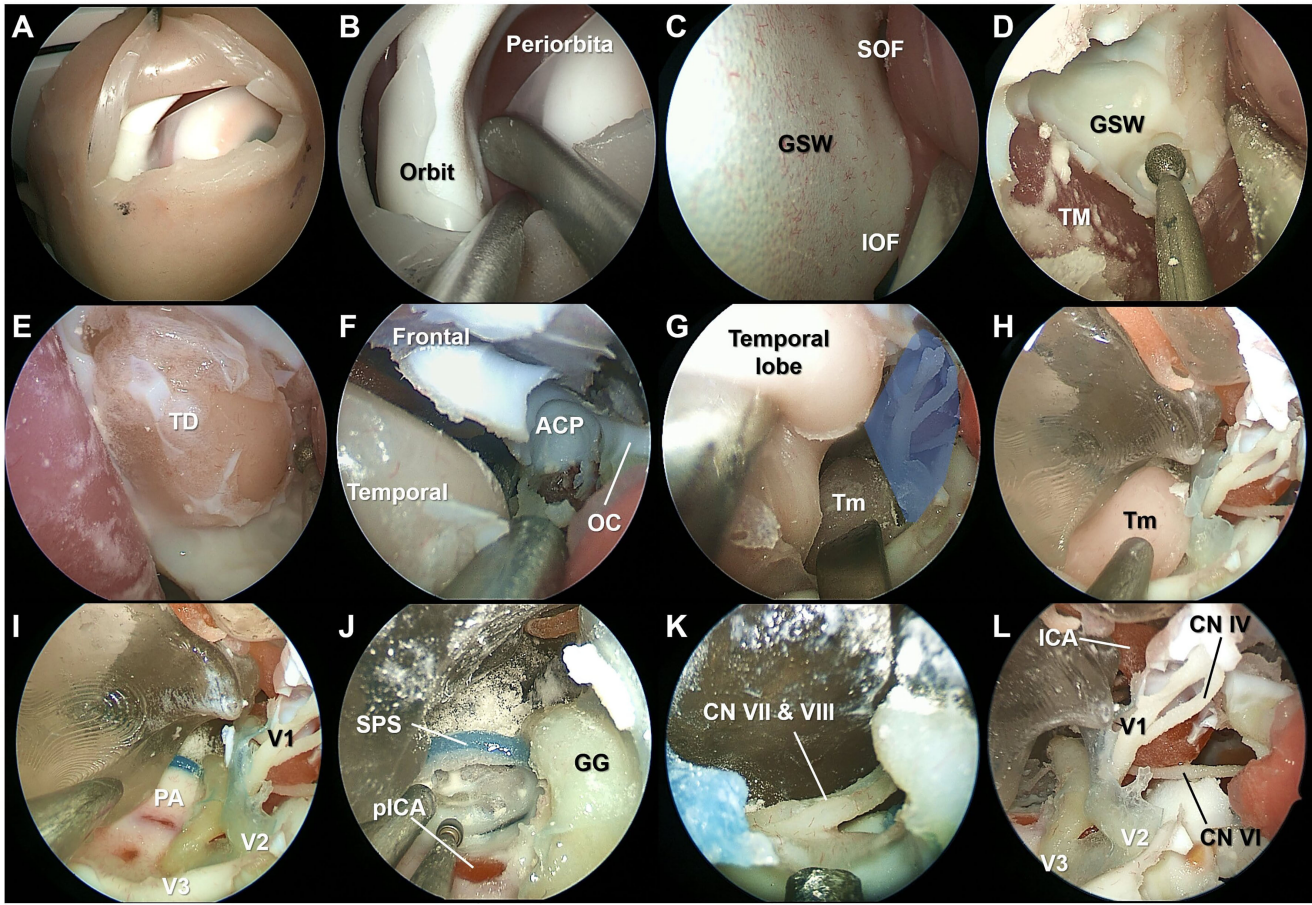
Stepwise dissection of the endoscopic transorbital surgery procedures using the simulation model. The superior eyelid incision was made on the simulation model **(A)**. Subperiosteal elevation of the periorbital was performed **(B)**. After identifying the superior (SOF) and inferior orbital fissure, the ocular surface of the greater sphenoidal wing was drilled to expose the middle cranial fossa **(C, D)**. The temporal dura was exposed, and the bone composing the lateral border of the SOF and maxillary strut was completely removed **(E, F)**. The lateral wall of the cavernous sinus (CS) (blue shaded area) was peeled off from the dura propria of the temporal lobe, and the tumor (Tm) lesion reconstructed from the patient with a trigeminal schwannoma was identified **(G, H)**. The ophthalmic branch (V1), maxillary branch (V2), and mandibular branch (V3) of the trigeminal nerve were identified after resection of the Tm **(I)**. Anterior petrosectomy was performed, and the seventh and eighth cranial nerves were identified **(J, K)**. The lateral wall of the CS was opened, and the neurovascular structures were identified **(L)**. TM, temporalis muscle; ACP, anterior clinoid process; OC, optic canal; PA, petrous apex; SPS, superior petrosal sinus; pICA, petrous segment of the internal carotid artery; GG, Gasserian ganglion.

The superior eyelid ETOS was performed on the same simulation model used in the EES procedures (**Figure 3**, **Video 2**). After subperiosteal dissection of the periorbita, the ocular surface of the greater sphenoid wing was drilled to expose the middle cranial fossa. The temporal dura was exposed, and the bone composing the lateral border of the superior orbital fissure was completely removed. The lateral wall of the CS was exposed, and a tumor lesion reconstructed from a patient with a trigeminal schwannoma was identified. The neurovascular structures within the CS were identified. The feasibility of anterior petrosectomy was evaluated, and the seventh and eighth cranial nerves were identified.

### Validation of the simulation model

The anatomical realism was scored as 4.0 ( ± 0.7) (**Table 2**). The participants agreed that the depth perception (4.4 [ ± 0.7]) and drilling sensation (4.7 [ ± 0.7]) were favorable. However, the color appearance (3.6 [ ± 0.7]) and soft tissue sensation (2.8 [ ± 0.8]) were scored low. The similarity of the model with the cadaver was rated as 3.4 ( ± 1.0). The participants rated the model as helpful in hand-eye coordination training (4.7 [ ± 0.5]) and improving surgical skills (4.6 [ ± 0.5]) for ESBS. All participants believed that the model was useful as educational support for trainees (4.7 [ ± 0.5]). From a task-based perspective, the model was rated as slightly more useful for the EES procedure (4.3 [ ± 0.6]) than the ETOS procedure (4.2 [ ± 0.8]). The utility of the model for surgical training of ESBS was rated as 4.6 ( ± 0.6). The participants agreed that the simulation model should be included in the resident training curriculum (4.6 [ ± 0.6]).

**Table 2:** Validation of the simulation model.

Categories	Questions	Mean 5-point Likert score (SD)
Anatomical realism	The anatomical structures are realistic.	4.0 ( ± 0.7)
	The depth perception is comparable to real surgery.	4.4 ( ± 0.7)
	The drilling sensation is comparable to a real human bone.	4.7 ( ± 0.7)
	The color appearance is close to real surgery.	3.6 ( ± 0.7)
	The soft tissue is realistic.	2.8 ( ± 0.8)
	Similarity to the cadaver.	3.4 ( ± 1.0)
Usefulness as a training tool	This model is useful for the hand-eye coordination training of ESBS.	4.7 ( ± 0.5)
	This model is useful for improving the surgical skills of trainees.	4.6 ( ± 0.5)
	This model can be used as an educational tool.	4.7 ( ± 0.5)
Task-based usefulness	EES procedures	4.3 ( ± 0.6)
	ETOS procedures	4.2 ( ± 0.8)
Overall reaction	This model is useful for ESBS training*	4.6 ( ± 0.6)
	I will recommend this model to my colleagues	4.7 ( ± 0.5)
	This model is useful for improving my surgical skills	4.5 ( ± 0.6)
	This model should be included in the resident training curriculum	4.6 ( ± 0.6)

*Only for participants who have at least one ESBS cadaver dissection course experience.

SD, standard deviation; ESBS, endoscopic skull base surgery; EES, endoscopic endonasal surgery; ETOS, endoscopic transorbital surgery.

**Figure 4:**
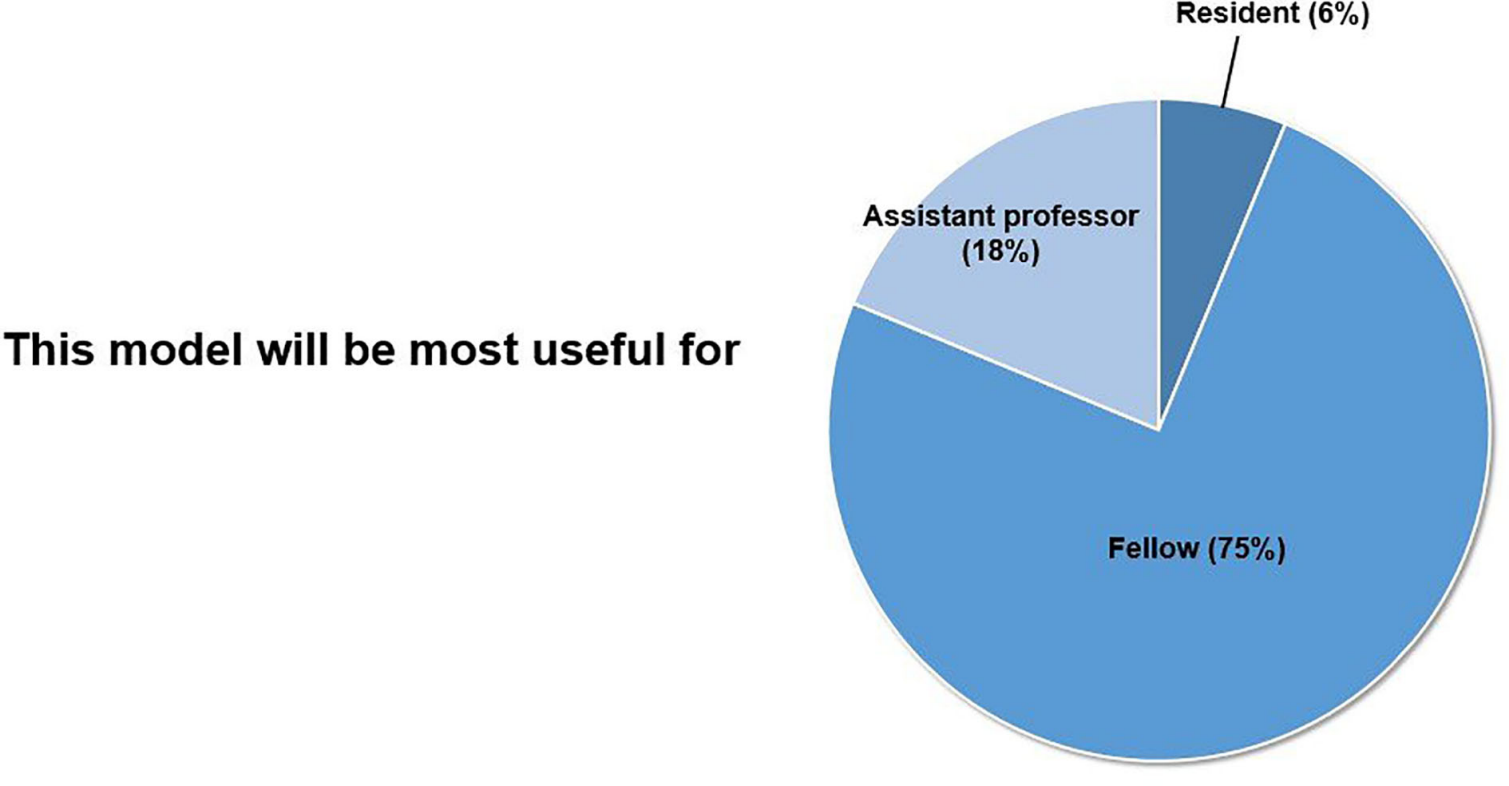
Usefulness as a training tool.

The participants reported that this simulation model would be most useful for the doctors in their fellowship (**Figure 4**). Thirty (93%) participants answered that they would attend this hands-on simulation course again.

## Discussion

The applications of 3D printing technology in neurosurgery are rapidly expanding, and 3D printed anatomical models are increasingly being used for education and surgical training (8, 13, 22–24). Surgical training using 3D printed simulation models allows practitioners and trainees to refine their surgical skills and anatomical knowledge before working in real clinical settings (6, 25). Providing additional education and training outside of the operating room is important for trainees to improve their skills and confidence. In this study, we designed and validated the efficacy of our 3D printed simulation model for ESBS training. All participants believed that the model is useful as an educational tool for trainees to improve their surgical techniques. The major purpose of the development of the 3D simulation model is to help trainees familiarize themselves with the unique viewing angle of the endoscope and the basic skills of endoscopic surgery. From our personal experience, trainees without basic skills in endoscope surgery tend to focus more on how to use the surgical instruments rather than on the anatomical features of the specimen or patient during the dissection. Meticulous bone drilling and fine dissection techniques within the small space are essential for surgeons performing ESBS. The depth perception of our model was comparable to a real surgery, and the model provided a realistic drilling sensation under an endoscopic environment. Although the color sensation and soft tissue feedback need to be improved, our model was nevertheless recognized as useful for participants to improve their surgical skills.

The use of the endoscopic technique in skull base surgery has been growing, and progressively more complex procedures are being performed (1, 12, 26). ETOS, the most recent field of neurosurgery, has been adopted for use in various skull base diseases (21, 26). Skull base surgery has little margin for error. To become proficient at this novel surgical procedure, surgeons must overcome the challenging ergonomics, unfamiliar anatomy, and high-stakes nature of surgery (27). Our model is the first 3D printed physical model for ETOS procedure simulation. We demonstrated the feasibility of performing the ETOS procedure in our model and validated the anatomical and surgical realism. A methodical training program using the realistic simulation models offers an attractive alternative to current training restricted to either lab-based cadaveric workshops or on-the-job training during live surgery (9, 10, 12, 28). The findings of the participants’ post-training subjective feelings also showed that all participants strongly agreed or agreed that they could benefit from ETOS simulation training using the model. With further validation of ongoing work, improvements in anatomy and haptic feedback can be achieved, which would lead to the simulation of various endoscopic surgical scenarios.

Overall, 3D models are powerful tools that can contribute to diverse fields of neurosurgery. Low cost and high fidelity have facilitated the wide adoption of 3D technology in neurosurgical education and training (8, 23, 29–33). From a patient-specific model perspective, our model was able to reproduce the tumor model. Such an individualized simulation model can be used in patient counseling, preoperative planning, and rehearsal of surgical procedures (34). Careful simulation before the real operation may contribute to improved patient safety and clinical outcomes by enhancing the surgical planning and procedural performance of the surgeon (25). The portability or digital transferability of the model is another advantage of the 3D model, which allows accessibility in multiple locations (13).

This study has several limitations. Firstly, the small number of participants is insufficient to fully validate the effectiveness of this model as a training tool. Second, the results were evaluated based only on the participants’ subjective feelings. Inaccuracies of self-reported experience are considerable. More objective scales should be developed for accurate verification of the model. Thirds, tactile feedback is one of the most important factors for decision-making during surgery. Our model lacks haptic feedback, such as pulsation, blood, or cerebrospinal fluid. Furthermore, the texture of the soft tissue was not fully replicated. Lastly, the impact of this simulation on actual surgery is unknown. Further validation of our models will be needed in the future. Efforts are currently underway to identify the best material to increase the fidelity of replicating the human body.

## Conclusion

In this study, we showed that high-resolution 3D printed skull base models for ESBS can be generated with high anatomical accuracy and acceptable haptic feedback. This model has the potential to be useful in education and surgical training for trainees who wish to learn and perform the EES and ETOS procedures. The simulation program of ESBS using this model may be supplemental or provide an alternative training platform to cadaveric dissection.

## Data availability statement

The original contributions presented in the study are included in the article/**Supplementary Material**. Further inquiries can be directed to the corresponding author.

## Ethics statement

The studies involving human participants were reviewed and approved by Samsung Medical Center Institutional Review Board. Written informed consent for participation was not required for this study in accordance with the national legislation and the institutional requirements.

## Author contributions

This study was designed and directed by D-SK and W-JL. D-SK, as the principal investigator, provided conceptual and technical guidance. W-JL analysed the data with YK, S-DH, T-HR, Y-SD, and YK. D-SK, S-DH, and C-KH interpreted the data and revised it critically for important intellectual content. The manuscript was written by W-JL and D-SK, with help from all authors. All authors contributed to the article and approved the submitted version.

## Conflict of interest

The authors declare that the research was conducted in the absence of any commercial or financial relationships that could be construed as a potential conflict of interest.

## Publisher’s note

All claims expressed in this article are solely those of the authors and do not necessarily represent those of their affiliated organizations, or those of the publisher, the editors and the reviewers. Any product that may be evaluated in this article, or claim that may be made by its manufacturer, is not guaranteed or endorsed by the publisher.

## Supplementary material

Supplementary Video 1 Simulation of the endoscopic endonasal surgery procedures using the three-dimensional printed skull base model.

Supplementary Video 2 Simulation of the endoscopic transorbital surgery procedures using the three-dimensional printed skull base model.

## References

[1] Almeida JP de Albuquerque LA Dal Fabbro M Sampaio M Medina R Chacon M. Endoscopic skull base surgery: Evaluation of current clinical outcomes. J Neurosurgical Sci (2019) 63(1):88–95. doi: 10.23736/s0390-5616.16.03386-5 26603533

[2] Zwagerman NT Zenonos G Lieber S Wang WH Wang EW Fernandez-Miranda JC. Endoscopic transnasal skull base surgery: Pushing the boundaries. J Neuro-oncol (2016) 130(2):319–30. doi: 10.1007/s11060-016-2274-y 27766473

[3] Locatelli D Pozzi F Turri-Zanoni M Battaglia P Santi L Dallan I. Transorbital endoscopic approaches to the skull base: Current concepts and future perspectives. J neurosurgical Sci (2016) 60(4):514–25. 27280546

[4] Patel CR Fernandez-Miranda JC Wang WH Wang EW. Skull base anatomy. Otolaryngol Clinics North America (2016) 49(1):9–20. doi: 10.1016/j.otc.2015.09.001 26614826

[5] Pojskić M Čustović O Erwin KH Dunn IF Eisenberg M Gienapp AJ. Microscopic and endoscopic skull base approaches hands-on cadaver course at 30: Historical vignette. World Neurosurg (2020) 142:434–40. doi: 10.1016/j.wneu.2020.07.064 32688034

[6] Suri A Patra DP Meena RK. Simulation in neurosurgery: Past, present, and future. Neurol India (2016) 64(3):387–95. doi: 10.4103/0028-3886.181556 27147144

[7] Xu JC Hanna G Fong BM Hsu FPK Cadena G Kuan EC. Ergonomics of endoscopic skull base surgery: A systematic review. World Neurosurg (2021) 146:150–5. doi: 10.1016/j.wneu.2020.11.026 33189918

[8] Thiong'o GM Bernstein M Drake JM. 3d printing in neurosurgery education: A review. 3D Print Med (2021) 7(1):9. doi: 10.1186/s41205-021-00099-4 33759067 PMC7989093

[9] Hsieh TY Cervenka B Dedhia R Strong EB Steele T. Assessment of a patient-specific, 3-dimensionally printed endoscopic sinus and skull base surgical model. JAMA Otolaryngol Head Neck Surg (2018) 144(7):574–9. doi: 10.1001/jamaoto.2018.0473 PMC6145784 29799965

[10] Zheng JP Li CZ Chen GQ Song GD Zhang YZ. Three-dimensional printed skull base simulation for transnasal endoscopic surgical training. World Neurosurg (2018) 111:e773–e82. doi: 10.1016/j.wneu.2017.12.169 29309974

[11] Zhang XD Li ZH Wu ZS Lin W Lin WJ Lin JC. A novel three-Dimensional-Printed paranasal sinus-skull base anatomical model. Eur Arch oto-rhino-laryngol (2018) 275(8):2045–9. doi: 10.1007/s00405-018-5051-z 29959564

[12] Narayanan V Narayanan P Rajagopalan R Karuppiah R Rahman ZA Wormald PJ. Endoscopic skull base training using 3d printed models with pre-existing pathology. Eur Arch oto-rhino-laryngol (2015) 272(3):753–7. doi: 10.1007/s00405-014-3300-3 25294050

[13] James J Irace AL Gudis DA Overdevest JB. Simulation training in endoscopic skull base surgery: A scoping review. World J Otorhinolaryngol Head Neck Surg (2022) 8(1):73–81. doi: 10.1002/wjo2.11 35619934 PMC9126166

[14] Colombo G Ferreli F Baram A Mercante G Riva M Di Maria A. Inferolateral transorbital endoscopic approach for spheno-orbital meningiomas. J Craniofacial Surg (2021) 33(3):e260–5. doi: 10.1097/scs.0000000000008062 34334747

[15] Gerges MM Godil SS Younus I Rezk M Schwartz TH. Endoscopic transorbital approach to the infratemporal fossa and parapharyngeal space: A cadaveric study. J Neurosurg (2019) 133(6):1948–59. doi: 10.3171/2019.7.Jns191743 31675695

[16] Kim EH Yoo J Jung IH Oh JW Kim JS Yoon JS. Endoscopic transorbital approach to the insular region: Cadaveric feasibility study and clinical application (Seven-005). J Neurosurg (2021) 135(4):1164–72. doi: 10.3171/2020.8.Jns202255 33482646

[17] Lim J Sung KS Kim W Yoo J Jung IH Choi S. Extended endoscopic transorbital approach with superior-lateral orbital rim osteotomy: Cadaveric feasibility study and clinical implications (Seven-007). J Neurosurg (2021) 137(1):18–31. doi: 10.3171/2021.7.Jns21996 34767525

[18] Lin BJ Hong KT Chung TT Liu WH Hueng DY Chen YH. Endoscopic transorbital transtentorial approach to middle incisural space: Preclinical cadaveric study. Acta Neurochirurgica (2019) 161(4):831–9. doi: 10.1007/s00701-019-03831-6 30758791

[19] López CB Di Somma A Cepeda S Arrese I Sarabia R Agustín JH. Extradural anterior clinoidectomy through endoscopic transorbital approach: Laboratory investigation for surgical perspective. Acta Neurochirurgica (2021) 163(8):2177–88. doi: 10.1007/s00701-021-04896-y 34110491

[20] Park HH Roh TH Choi S Yoo J Kim WH Jung IH. Endoscopic transorbital approach to mesial temporal lobe for intra-axial lesions: Cadaveric study and case series (Seven-008). Oper Neurosurg (Hagerstown) (2021) 21(6):E506–e15. doi: 10.1093/ons/opab319 34528091

[21] Yoo J Park HH Yun IS Hong CK. Clinical applications of the endoscopic transorbital approach for various lesions. Acta Neurochirurgica (2021) 163(8):2269–77. doi: 10.1007/s00701-020-04694-y 33394139

[22] Badash I Burtt K Solorzano CA Carey JN. Innovations in surgery simulation: A review of past, current and future techniques. Ann Transl Med (2016) 4(23):453. doi: 10.21037/atm.2016.12.24 28090509 PMC5220028

[23] Blohm JE Salinas PA Avila MJ Barber SR Weinand ME Dumont TM. 3d printing in neurosurgery residency training: A systematic review of the literature. World Neurosurg (2021) 161:111–22. doi: 10.1016/j.wneu.2021.10.069 34648984

[24] Aimar A Palermo A Innocenti B. The role of 3d printing in medical applications: A state of the art. J Healthc Eng (2019) 2019:5340616. doi: 10.1155/2019/5340616 31019667 PMC6451800

[25] Rehder R Abd-El-Barr M Hooten K Weinstock P Madsen JR Cohen AR. The role of simulation in neurosurgery. Child's Nervous System (2016) 32(1):43–54. doi: 10.1007/s00381-015-2923-z 26438547

[26] Verillaud B Bresson D Sauvaget E Mandonnet E Georges B Kania R. Endoscopic endonasal skull base surgery. Eur Ann Otorhinolaryngol Head Neck Dis (2012) 129(4):190–6. doi: 10.1016/j.anorl.2011.09.004 22321910

[27] Davids J Manivannan S Darzi A Giannarou S Ashrafian H Marcus HJ. Simulation for skills training in neurosurgery: A systematic review, meta-analysis, and analysis of progressive scholarly acceptance. Neurosurg Rev (2021) 44(4):1853–67. doi: 10.1007/s10143-020-01378-0 PMC8338820 32944808

[28] Hochman JB Rhodes C Wong D Kraut J Pisa J Unger B. Comparison of cadaveric and isomorphic three-dimensional printed models in temporal bone education. Laryngoscope (2015) 125(10):2353–7. doi: 10.1002/lary.24919 26256951

[29] Mooney MA Cavallo C Zhou JJ Bohl MA Belykh E Gandhi S. Three-dimensional printed models for lateral skull base surgical training: Anatomy and simulation of the transtemporal approaches. Oper Neurosurg (Hagerstown) (2020) 18(2):193–201. doi: 10.1093/ons/opz120 31172189

[30] McGuire LS Fuentes A Alaraj A. Three-dimensional modeling in training, simulation, and surgical planning in open vascular and endovascular neurosurgery: A systematic review of the literature. World Neurosurg (2021) 154:53–63. doi: 10.1016/j.wneu.2021.07.057 34293525

[31] Huang X Liu Z Wang X Li XD Cheng K Zhou Y. A small 3d-printing model of macroadenomas for endoscopic endonasal surgery. Pituitary (2019) 22(1):46–53. doi: 10.1007/s11102-018-0927-x 30506234 PMC6373287

[32] Huang X Fan N Wang HJ Zhou Y Li X Jiang XB. Application of 3d printed model for planning the endoscopic endonasal transsphenoidal surgery. Sci Rep (2021) 11(1):5333. doi: 10.1038/s41598-021-84779-5 33674649 PMC7935876

[33] Chien WW da Cruz MJ Francis HW. Validation of a 3d-printed human temporal bone model for otology surgical skill training. World J Otorhinolaryngol Head Neck Surg (2021) 7(2):88–93. doi: 10.1016/j.wjorl.2020.12.004 33997717 PMC8103535

[34] Blohm JE Salinas PA Avila MJ Barber SR Weinand ME Dumont TM. Three-dimensional printing in neurosurgery residency training: A systematic review of the literature. World Neurosurg (2022) 161:111–22. doi: 10.1016/j.wneu.2021.10.069 34648984

